# Evaluation of the range shifter model for proton pencil‐beam scanning for the Eclipse v.11 treatment planning system

**DOI:** 10.1120/jacmp.v17i2.5798

**Published:** 2016-03-08

**Authors:** Witold Matysiak, Daniel Yeung, Roelf Slopsema, Zuofeng Li

**Affiliations:** ^1^ University of Florida Proton Therapy Institute Jacksonville FL USA

**Keywords:** particle therapy, pencil‐beam scanning, treatment planning systems

## Abstract

Existing proton therapy pencil‐beam scanning (PBS) systems have limitations on the minimum range to which a patient can be treated. This limitation arises from practical considerations, such as beam current intensity, layer spacing, and delivery time. The range shifter (RS) — a slab of stopping material inserted between the nozzle and the patient — is used to reduce the residual range of the incident beam so that the treatment ranges can be extended to shallow depths. Accurate modeling of the RS allows one to calculate the beam spot size entering the patient, given the proton energy, for arbitrary positions and thicknesses of the RS in the beam path. The Eclipse version 11 (v11) treatment planning system (TPS) models RS‐induced beam widening by incorporating the scattering properties of the RS material into the V‐parameter. Monte Carlo simulations with Geant4 code and analytical calculations using the Fermi‐Eyges (FE) theory with Highland approximation of multiple Coulomb scattering (MCS) were employed to calculate proton beam widening due to scattering in the RS. We demonstrated that both methods achieved consistent results and could be used as a benchmark for evaluating the Eclipse V‐parameter model. In most cases, the V‐parameter model correctly predicted the beam spot size after traversing the RS. However, Eclipse did not enforce the constraint for a nonnegative covariance matrix when fitting the spot sizes to derive the phase space parameters, which resulted in incorrect calculations under specific conditions. In addition, Eclipse v11 incorrectly imposed limits on the individual values of the phase space parameters, which could lead to incorrect spot size values in the air calculated for beams with spot sigmas <3.8 mm. Notably, the TPS supplier (Varian) and hardware vendor (Ion Beam Applications) inconsistently refer to the RS position, which may result in improper spot size calculations.

PACS number(s): 87.53.Jw, 87.53.Kn, 87.55.kd, 87.56.‐v

## I. INTRODUCTION

The number of proton therapy centers has grown rapidly in the last decade. As of December 2013, over 122,000 patients have been treated at 42 centers worldwide using energetic charged particles.^(1)^ While most treatments use the passive scattering technique, newer centers are adopting the more advanced pencil‐beam scanning (PBS) technique, which reduces the out‐of‐the‐field dose and given sufficiently small spot size can also achieve better dose conformality. In passive scattering, the range of the proton beam is modulated to create the spread‐out Bragg peak (SOBP).^2^, ^3^ Ridge filters or, more commonly, rotating modulator wheels,^(4)^ are used to create treatment fields of variable modulation depths that can extend to the skin surface. In the PBS mode, the proton beam (with an initial momentum spread determined by the beam production method and beam line transport properties) arrives in the treatment nozzle at the energy matching the depth of the current layer to be irradiated. The number of layers, their depths, relative intensities, and the spacing between layers (dictated by the width of the pristine peaks) are optimized to yield a flat SOBP of a given modulation. Since the absolute width of the pristine peak decreases with decreasing energy, lower energy beams require a greater number of layers, thus longer delivery times. An additional difficulty arises in fixed‐energy production systems (i.e., cyclotrons) where the efficiency of the energy selection system gradually decreases with decreasing range.

The range shifter (RS) — a slab of material inserted in the beam path — provides a practical solution to enable treatment in shallow depths. At RS exit, the residual range of the beam is decreased, while both the energy spread (range straggling) and lateral spread (MCS) are increased. To minimize the spot size of the broadened pencil beam, the RS should be positioned as close as possible to the skin. For the IBA (Ion Beam Applications, Louvain‐la‐Neuve, Belgium) system with the universal nozzle, this can be achieved with a travelling snout on which the range shifter (Lexan) is mounted. As a result, one must ensure that the treatment planning system (TPS) can accurately model the beam size downstream of the RS, which accounts for the variable positions of the snout.

We used two independent methods, Monte Carlo simulations using Geant4 code and analytical calculations using the Fermi‐Eyges theory^(5)^ with Highland approximation of multiple Coulomb scattering,^(6)^ to evaluate the accuracy of the RS model implemented in the Eclipse v.11 TPS.

## II. MATERIALS AND METHODS

### A. The Fermi‐Eyges description of beam propagation

The propagation of a proton beam can be described using the Fermi‐Eyges theory. Gottschalk^(7)^ provided a description of the theory applied to particle therapy, as well as a theoretical framework for experimental determination of the phase space parameters. For consistency with the nomenclature used in Eclipse beam configuration documents, we referred to the phase space parameters as “A,” “B,” and “C,” which correspond to the 2nd, 1st, and 0th order Fermi‐Eyges scattering moments, respectively. With the convention that the beam propagates along the z‐axis, parameter “A” designates the variance of proton positions projected on one of the orthogonal planes (xz or yz); similarly, parameter “C” describes the variance in projections of the momentum directions; and parameter “B” signifies the covariance between “A” and “C.” As the particles propagate along the beam axis, the phase space parameters evolve according to Eq. set (A. 1) in Appendix A. Based on this set of equations, all three phase space parameters can be calculated at any position along the beam propagation direction.

### B. The beam propagation model in Eclipse and the V‐parameter

The model of beam propagation in Eclipse is partially based on the Fermi‐Eyges theory. However, instead of relying on the nominal values for the RS properties (thickness, density, and radiation length), the scattering power of the RS is determined indirectly during the TPS commissioning process. The model assumes that the scattering power is inversely proportional to the kinematic factor with an energy‐independent proportionality constant called the V‐parameter, which describes the scattering properties of the RS material: $\vartheta =\frac{\sqrt{V}}{\text{pv}}$. Note that, if the user chooses to work with the cylindrical Gaussian model to describe the beam spot size, the right side of the formula must be divided by $\sqrt{2}$.

**Figure 1:**
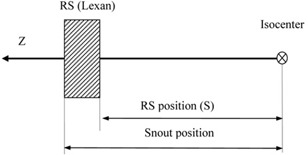
Geometry relating the positions of the snout and the range shifter.

The V‐parameter is determined from a pair of measurements: with and without the RS in place. When the RS is inserted into the beam path, the parameters on the left side of equation set 1 change only due to the integral expressions on the right side, and the change in the phase space parameters can be calculated as (private communication with Varian Medical Systems: S. Siljamaki, http://Sami.Siljamaki@varian.com, May 5, 2014) presented in the Eq. set (A.6) in Appendix A. The only two free parameters in this equation set are the water‐equivalent thickness of the RS (L) and the distance in air to the reference plane where the $A_{0},B_{0}$, and $C_{0}$ parameters are defined (S in Fig. 1).

The geometrical conventions implemented in the Eclipse by default assume that the snout position, the distance from the isocenter to the downstream side of the device without consideration of variable length elements as defined by the DICOM standard (Digital Imaging and Communications in Medicine Standard Supplement 102: Radiotherapy Extensions for Ion Therapy, National Electrical Manufacturers Association, Rosslyn, VA), coincides with the downstream surface of the RS. This assumption makes it unnecessary to measure the physical thickness of the RS since only the water‐equivalent thickness (L) is needed for the V‐parameter model. Consequently, Eclipse beam configuration does not require input of this parameter. However, in the IBA system, the snout position is defined with respect to the downstream face of the aperture at which the RS is mounted. Therefore, the RS upstream surface coincides with the snout position (Fig. 1). Such a convention is more flexible because (e.g., it allows one to use patient‐specific apertures and the RS simultaneously). Hitachi (Hitachi Ltd, Tokyo, Japan), a proton therapy system vendor who equipped the MD Anderson Cancer Center, uses the downstream surface of the snout rather than the downstream face of the aperture, as in the IBA system, to define the snout position. With the clinically commissioned RS of 3.5 cm water‐equivalent thickness, the downstream surface of the RS extends 3.5 cm downstream of the snout position and is used to define the RS position in Eclipse, whereas the downstream surface of the aperture is located 3.0 cm upstream of the so‐defined snout position (private communication with MD Anderson Cancer Center: Ron Zhu, xrzhu@mdanderson.org, Sept. 8, 2015). The different interpretations of the term “RS position” must be addressed in the commissioning process by introducing an offset equal to the physical thickness of the RS between the snout position and the downstream surface position of the RS. In Eclipse v11, this is accomplished with a translation table that calculates the downstream position of the RS based on the snout position.

Given the two sets of phase space measurements — with and without the RS in place — acquired during the commissioning process, the V‐parameter can be extracted from any one of the formulae in Eq. set (A.6) in Appendix A. To account for the contribution of large angle scattering events to the broadening of the pencil‐beam profile, the V‐parameter is further modified according to the Lynch and Dahl formula.^(8)^

Eclipse calculates the V‐parameter from all three equations of Eq. set (A.6) using available experimental data for all proton energies acquired in machine commissioning and uses the average value for subsequent calculations. By entering the corresponding values of the two free parameters — L and S in the equation — the user can calculate the phase space parameters for any given position and thickness of the RS using Eq. set (A.7) found in Appendix A.

We evaluated the accuracy of the calculated phase space parameters for different RS positions. Typically, a RS with only one water‐equivalent thickness, which corresponds to the minimum range that the accelerator system can achieve in a patient, is clinically commissioned in the TPS. The RS then allows treatment depths to extend from the minimum range up to the patient's surface. Consequently, we did not investigate the accuracy of the V‐parameter model for different RS thicknesses. As stated earlier, Monte Carlo simulations with the Geant4^(9)^ code and analytical calculations using the Fermi‐Eyges theory incorporating the Highland approximation of the Molière multiple Coulomb scattering theory^(6)^ were used as benchmarks.

### C. Initial phase space parameters

An experimental dataset acquired with the Lynx scintillation screen (IBA Dosimetry GmbH, Schwarzenbruck, Germany) in gantry room 4 of the Westdeutsches Protontherapiezentrum Essen (WPE) was used to commission a PBS beam model for this study. The dataset consisted of two sets of measurements, one taken with the open beam (i.e., no RS) and another with the RS inserted in the beam path with the downstream surface 36.5 cm from the isocenter. In order to obtain the phase space parameters in both orthogonal directions X and Y at the reference position z=0, Eq. (A.1a) was fitted to the beam spot profiles σ as a function of the beam propagation direction “Z.” The fitting routine was subject to the constraint that the determinant of the covariance matrix $\left(A_{0}C_{0}-{B_{0}}^{2}\right)$ had to be positive^(10)^ (i.e., the beam envelope could only take positive values). These phase space parameters were used to define the initial beam condition in Monte Carlo simulations and Fermi‐Eyges theory calculations.

### D. Phase space parameters in Eclipse

**Figure 2:**
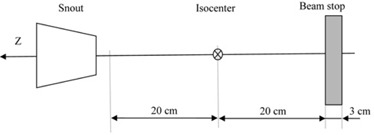
Schematic of a phantom built in Eclipse to extract phase space parameters.

In order to evaluate the in‐air spot sizes calculated by Eclipse based on the commissioned PBS beam data from WPE, we built a phantom image set using 231 planes separated by 2 mm, so that the total length of the phantom in the direction of the beam propagation was 46 cm. Each plane consisted of a 512×512 pixel matrix with 0.39 mm resolution in both orthogonal directions. All pixels were assigned a CT value of ‐1000 HU (corresponding to the relative proton stopping power of zero) except for a distal slab of 3 cm in thickness, which had an arbitrary CT value of 2800 HU (corresponding to the relative proton stopping power of 10), which served as a beam stop (Fig. 2). This arrangement allowed for a free drift region to extend symmetrically between ‐20 cm and +20 cm around the isocenter. The dose‐grid calculation resolution was set at 1 mm to minimize rounding errors. For each of the five arbitrarily chosen proton energies spanning the clinically relevant ranges (105, 140, 160, 225, and 226.7 MeV), a single‐layer PBS plan was created and the spot map was manually edited to remove all but the center spot. Three arbitrarily selected RS positions for each of the energies were studied (17.5 cm, 26.5 cm, and 36.5 cm), as well as an additional (open‐beam) dataset without the RS. The open‐beam data and the dataset with the RS position of 36.5 cm were used to commission the RS in Eclipse.

For any RS position other than 36.5 cm, Eclipse calculates the phase space parameters using the V‐parameter model. The dose plans were exported in DICOM‐RT Dose format (radiotherapy extension of the Digital Imaging and Communications in Medicine standard) and a MATLAB^(11)^ program was used to fit two‐dimensional Gaussian functions to the dose planes at selected “z” positions around the isocenter between ‐20 cm and +20 cm in 5 cm increments. The spatial resolution of the RT Dose files was lower than the 1 mm dose grid resolution used for all calculations, thereby ensuring that spot sizes extracted from the fitting procedure were not affected by the discretization error.

### E. Monte Carlo model

Geant4 (ver. 9.6 p2) is a well‐established Monte Carlo simulation code^12^, ^13^, ^14^, ^15^ for proton therapy applications. The toolkit provides flexible selection of physics processes, but the user is ultimately responsible for choosing the correct modules for the “physics list.” Our group previously verified the suitability of the multiple Coulomb scattering model implemented in Geant4 for RS simulations in the clinically relevant proton energy range.^(16)^ Because we found satisfactory agreement between the simulated and experimental data, we are confident in the results that we obtained in this study.

**Figure 3:**
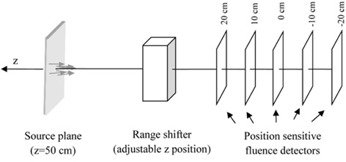
Geometry used for the Monte Carlo simulation with Geant4.

The simulation environment (Fig. 3) consisted of a source located upstream of the RS investigated in the study, at an arbitrary position of z=50 cm from the isocenter. To model the source, the phase space parameters defined at the reference position (isocenter, z=0 cm) were extracted from the measured data. In order to transform the phase space parameters for the nominal source position at z=50 cm (Δz=50 cm), Eq. set (A.1) was employed, with all the integral terms set to 0 (free drift condition, Eq. set (A.4)).

The source was defined as a surface perpendicular to the direction of the beam propagation axis. The starting positions $\left(x_{s},y_{s}\right)$, as well as momentum directions $\left(\theta_{\text{xs}},\theta_{y\theta }\right)$ of the source protons, were sampled from two 2D Gaussian distributions (Eq. (A.5)), independently in X and Y directions.

The RS was modeled as a 6.5 cm thick slab of Lexan (polycarbonate) as used in the actual IBA treatment nozzle. The position of the RS (downstream surface) was varied from 36.5 cm (the reference position) to 17.5 cm (close to the most proximal position allowed by the TPS for this phantom geometry without colliding with the snout). The position‐sensitive fluence detectors recorded x and y positions of individual protons crossing the detector surface. A MATLAB (MathWorks, Natick, MA) routine was used offline to fit the Gaussian distribution to the fluence profiles to extract the beam spot sizes as a function of the position along the beam axis, σ(z). Each simulation required 200,000 source protons to accumulate profiles of sufficient statistical quality. This number of source protons was considered adequate to obtain statistically valid results for the following two reasons: 1) almost each source proton contributed to the result being tallied, and 2) non‐Gaussian effects of the MCS, as well as production of secondary particles, were not included in this analysis.

### F. Analytical calculations

As a second reference, we performed analytical calculations based on the Fermi‐Eyges theory. The same set of proton energies and RS positions were used in this calculation as in the Monte Carlo simulation. For the scattering power, the Highland approximation of the Molière multiple scattering theory^(6)^ was employed, which for radiotherapy‐energy protons agrees with the Molière theory within 6%.^(17)^ By combining (A.1a), (A.1b), the phase space parameters at the isocenter with the RS inserted can be calculated as presented in Eq. set (A.3). We used the proton energy‐range relation in Lexan provided by The National Institute of Standards and Technology^(18)^ and, following the procedure described by Gottschalk et al.,^(17)^ we excluded the logarithmic correction factor from all integrals.

## III. RESULTS & DISCUSSION

### A. Validation of the reference methods

The first objective was to validate the two reference methods, Monte Carlo simulations with Geant4 and analytical calculations using the Fermi‐Eyges theory, against experimentally measured data. The experimental setup consisted of the IBA dedicated nozzle with an attached Lynx scintillation screen using an adjustable positioning frame; thus, the location of the screen could be varied between −20 cm and +20 cm with respect to the isocenter along the beam central axis. Two‐dimensional beam profiles were acquired with a resolution of 0.5 mm in both transverse directions for selected positions of the scintillation screen in steps of 10 cm. Using the procedure described in the Materials & Methods section, the beam phase space parameters for three selected proton energies spanning the clinically relevant range were calculated and used as input for the two reference methods. In the next step, the 6.5 cm Lexan RS was inserted in the beam path at the position of 36.5 cm (downstream face) with respect to the isocenter, and the two‐dimensional profiles were again acquired with the Lynx detector.

Given the phase space of the open beam (i.e., without the RS in place), the two reference methods were used to predict the evolution of the beam profile with the RS in place (Fig. 3).

**Figure 4:**
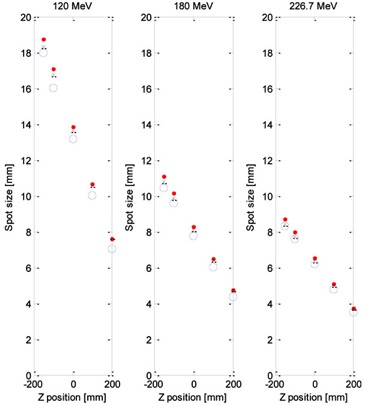
Comparison of experimentally measured spot sizes (open circles) with those obtained analytically using the Fermi‐Eyges theory (dots) and Geant4 Monte Carlo simulations (crosses) for selected proton energies.

Both reference methods correctly predict the beam spot size after traversing the RS for all three proton energies (Fig. 4). The greatest difference between the experimentally obtained spot sizes and those predicted by the reference methods was observed for the lowest proton energy (120 MeV) and was below 1 mm. For the two higher energies (180 MeV and 226.7 MeV), the experimental results agreed with the calculations to a fraction of a millimeter, which gave us confidence in our methodology.

### B. Spot sizes calculated in Eclipse using the V‐parameter model vs. reference methods

We compared the spot sizes calculated by the Eclipse V‐parameter model with the two reference methods. The initial phase space for the reference methods was derived from the Eclipse single‐layer central spot plans without the RS in place. Next, we used the reference methods to compute spot sizes around the isocenter with the RS in the beam path at three selected positions and compared them with the spot sizes calculated by Eclipse for the corresponding RS positions.

### C. Presentation of the results below and above the proton energy of 165 MeV

**Figure 5:**
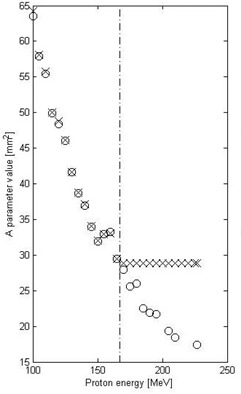
Value of the A parameter without the RS in the beam derived by Eclipse from the experimental data (open circles) and the A parameter used by Eclipse for calculations (crosses). Note that for the region above the vertical line at 165 MeV the values were affected by the software “bug” in Eclipse.

The presentation of the comparisons between spot sizes obtained using the V‐parameter model calculations and those from the reference methods was partitioned into two proton energy regions: below and above 165 MeV. This division was introduced due to a known software “bug” (private communication with Varian Medical Systems: S. Siljamaki, Sami.Siljamaki@varian.com, May 5, 2014) present in Eclipse v11 and corrected in subsequent releases, which erroneously imposed limits on the phase space parameters (A, B, and C) for higher energies if the corresponding in‐air spot sigmas at isocenter dropped below ∼3.8 mm. Figure 5 presents two sets of the A‐parameter data ($A=2\sigma_{\text{iso}}^{2}$, where $\sigma_{\text{iso}}$ is the spot size at the isocenter) internally calculated by the Eclipse beam configuration engine as a function of proton energy. The first set (open circles) presents the data directly derived by Eclipse from the experimental data uploaded to the beam configuration section, and the second set presents the data used for dose calculations (crosses). Due to the flawed implementation, the values of the A‐parameter for proton energies above 165 MeV were set to a constant value, whereas the values of the A‐parameter below 165 MeV were unaffected. Parameters B and C were similarly affected.

#### C.1 Below 165 MeV

**Figure 6:**
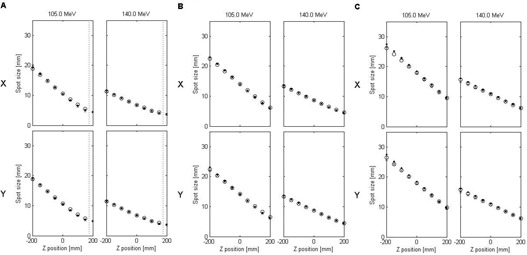
Comparison of the spot sizes in air calculated by Eclipse (crosses), simulated with Geant4 (open circles), and calculated analytically using the Fermi‐Eyges theory (dots) for RS positions: 17.5 cm (a), 26.5 cm (b), and 36.5 cm (c) and proton energies below 165 MeV. The dashed vertical line in (a) marks the position of the downstream face of the RS. Fitting uncertainties, as well as statistical uncertainties of Monte Carlo simulations, were too small to be shown on the graph.

Figures 6(a) to (c) show the comparison of spot sizes with the RS in place at three arbitrarily chosen locations (17.5 cm, 26.5 cm, and 36.5 cm) and proton energies below 165 MeV for three conditions: 1) calculated by Eclipse, 2) simulated with the Geant4 code, and 3) calculated analytically using the Fermi‐Eyges theory. The spot sizes calculated by the two reference methods closely matched those calculated by the Eclipse V‐parameter model for all studied RS positions. All three methods were in good agreement in modeling the spot size changes at different RS positions.

#### C.2 Above 165 MeV

**Figure 7:**
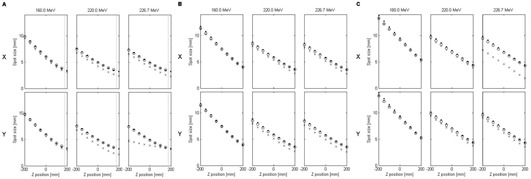
Comparison of the spot sizes in air calculated by Eclipse (crosses), simulated with Geant4 (open circles), and calculated analytically using the Fermi‐Eyges theory (dots) for positions of the RS: 17.5 cm (a), 26.5 cm (b), and 36.5 cm (c) and proton energies above 165 MeV. The dashed vertical line in (a) marks the position of the downstream face of the RS. Fitting uncertainties, as well as statistical uncertainties of Monte Carlo simulations, were too small to be shown on the graph.

Figures 7(a) to (c) show similar comparisons but for proton energies above 165 MeV. Both reference methods show spot sizes systematically higher than those calculated by the Eclipse V‐parameter model. The results were consistent with the known software flaw in Eclipse that set individual limits on the phase space parameters.

**Figure 8:**
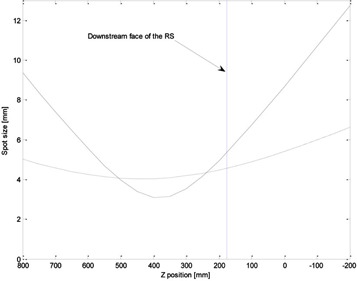
Spot size in air for the proton energy of 226.7 MeV in Y direction evaluated from Eclipse internal phase space, with the RS in place (solid line) and without the RS in place (dotted line).

In addition, the spot size envelope shows significant departures from that calculated by the reference methods in two cases:

- For the proton energy of 226.7 MeV in the Y direction and the RS position of 17.5 cm (19.0 cm from the reference position), the Eclipse calculation erroneously discarded the correction applied to the phase space by the V‐parameter model and produced a user interface warning that the spot size without the RS block was higher than with the RS block. Figure 8 illustrates the spot size evaluated based on both sets of phase space parameters, with and without the RS in place for this proton energy and direction. Downstream of the RS, the spot size with the RS in place is always bigger than the spot size with the open beam. The erroneous comparison of the spot sizes performed by Eclipse was done by extrapolating the beam envelope corrected for the presence of the RS upstream of the RS surface (z>17.5 cm). The calculated spot size with the RS in place (solid line in Fig. 8) is indeed lower than that without the RS in the beam for “z” positions between ∼50 and 25 cm, but this comparison is unphysical. As a result of discarding the phase space corrections, the beam envelope was considerably different than that predicted by Monte Carlo simulations and analytical calculations.
- For the proton energy of 226.7 MeV in the X direction and the RS position of 36.5 cm (reference position), the spot sizes reported by Eclipse were smaller by approximately 5 mm than those calculated using the Fermi‐Eyges theory and simulated by Geant4. This discrepancy was due to an inconsistency in Eclipse's internal fitting routine for deriving the phase space parameters. When fitting experimental data to the phase space model (Eq. (A.1)), Eclipse did not ensure that the determinant of the covariance matrix (i.e., minimum spot size in free drift) was nonnegative. Furthermore, the program did not verify if the phase space parameter values interpolated for proton energies other than the experimental values might also become nonpositive. In the case of the calculation at 226.7 MeV in the X direction, the RS was located at the reference position; thus, no V‐parameter corrections were necessary, and the uncorrected phase space parameters were used in calculations. At this point, Eclipse calculated the determinant, recognized that it was negative (although it did not produce a user interface warning), and adjusted the A, B, and C parameters until the determinant was no longer negative, which in turn led to an incorrect calculation of the spot sizes.

## IV. CONCLUSIONS

The V‐parameter model implemented in the Eclipse v11 TPS is based on the Fermi‐Eyges theory and Gaussian approximation of multiple Coulomb scattering. However, rather than relying on the nominal properties of the RS material, the model derives scattering characteristic of the material from experimental data used in TPS commissioning. The results obtained with the model are consistent with those obtained using the two reference methods (MC simulation with Geant4 and analytical calculations using the FE theory) and correctly predict the evolution of spot sizes in air with varying RS position even when the RS is positioned away (19 cm maximum in our study) from the reference position. However, the team commissioning pencil‐beam scanning must understand the implementation details to avoid potential pitfalls.

The “bug” limiting phase space parameters most likely arose because small spot sizes (<3.8 mm) were not anticipated by the software development team due to technical limitations of proton therapy equipment at that time. The considerable improvements that have been made in particle therapy technique (e.g., the dedicated pencil‐beam scanning nozzle) caused what may have been implemented as a safety feature to resurface as a software flaw. For proton energies not affected by this deficiency (energies <165 MeV in the dataset), the difference between the spot sizes calculated by Eclipse and those calculated using the reference methods was below 5%. For proton energies above 165 MeV, Eclipse's calculated spot sizes were lower than those of the reference methods, with a maximum difference around the isocenter of approximately 20%. The clinical effect of the “bug” in Eclipse is visible in the most distal energy layer, as well as all proximal layers. Since the spot size modeled by Eclipse is bigger than the true spot size for all proton energies where the true spot size measured at isocenter in air is lower than ∼3.8 mm (in our dataset this proton energy is ∼165 MeV), the true uniformity of the most distal layer may be worse than that calculated by the plan. What is more, although the “bug” is limited to energy layers higher than 165 MeV, its effect may be visible in the entire plan. This is due to the algorithm implemented in Eclipse that sets the spot spacings in all layers of the plan using the calculated spot spacing for the most distal layer. As a result, spatial arrangement of the spots in all proximal layers will be affected.

In addition, discrepancies have been identified at the maximum proton energy (226.7 MeV) where in some cases the phase space was not correctly propagated. This is due to the implementation flaw that set individual limits on the phase space parameters and a shortcoming of the algorithm that did not ensure nonnegativity of the covariance matrix (minimum spot size in free drift) in the fitting procedure.

Finally, the commissioning team should ensure that the RS position definition is used consistently, as the TPS vendor and the equipment manufacturer may use different geometric conventions.

## ACKNOWLEDGMENTS

The authors would like to thank Varian Medical Systems for discussions and sharing internal documentation regarding implementation of the RS model in the Eclipse TPS. We also thank Kate Casey‐Sawicki, MA, and the research staff in the UF Department of Radiation Oncology for editing and preparing this manuscript for publication.

## COPYRIGHT

This work is licensed under a Creative Commons Attribution 4.0 International License.

## APPENDICES

Appendix A: **Calculation of the Phase Space Parameters.**

*1. According to the Fermi‐Eyges Theory*

(A.1a) $$A(z)=C_{0}z^{2}+2B_{0}z+A_{0}+\int_{0}^{z}{\left(z-z^{\prime }\right)}^{2}\vartheta^{2}(z^{\prime })$$

(A.1b) $$B(z)=C_{0}z+B_{0}+\int_{0}^{z}{\left(z-z^{\prime }\right)}^{2}\vartheta^{2}(z^{\prime })$$

(A.1c) $$C(z)=C_{0}+\int_{0}^{z}\vartheta^{2}(z^{\prime })dz^{\prime }$$

where the subscripted variables refer to the phase space parameters determined at a reference position (e.g., z=0), and θ is the scattering power of the medium. The latter can be calculated using the Highland formula:

(A.2) $$\vartheta =\frac{14.1MeV}{pv}\sqrt{\frac{L}{L_{R}}}\left(1+\frac{1}{9}\text{log}\left(\frac{L}{L_{R}}\right)\right)$$

where *L* is the mass thickness of the RS, $L_{R}$ is the radiation length of polycarbonate (Lexan), and *pv* is the kinematic factor (i.e., the product of the particle momentum and velocity).

Formulae used to calculate the phase space parameters with the RS in the beam path of water equivalent thickness L at position S:

(A.3a) $$A_{RS}=A_{0}+{\left(14.1Mev\left(1+\frac{1}{9}\text{log}\left(\frac{L}{L_{R}}\right)\right)\right)}^{2}\int_{0}^{\frac{L}{\rho }}{\left(\frac{L}{\rho }+S-z^{\prime }\right)}^{2}\frac{\rho }{L_{R}}\frac{1}{{\left(pv(z^{\prime })\right)}^{2}}dz^{\prime }$$

(A.3b) $$B_{RS}=B_{0}+{\left(14.1Mev\left(1+\frac{1}{9}\text{log}\left(\frac{L}{L_{R}}\right)\right)\right)}^{2}\int_{0}^{\frac{L}{\rho }}\left(\frac{L}{\rho }+S-z^{\prime }\right)\frac{\rho }{L_{R}}\frac{1}{{\left(pv(z^{\prime })\right)}^{2}}dz^{\prime }$$

(A.3c) $$C_{RS}=C_{0}+{\left(14.1Mev\left(1+\frac{1}{9}\text{log}\left(\frac{L}{L_{R}}\right)\right)\right)}^{2}\int_{0}^{\frac{L}{\rho }}\frac{\rho }{L_{R}}\frac{1}{{\left(pv(z^{\prime })\right)}^{2}}dz^{\prime }$$

Free drift condition of the Fermi‐Eyges theory used to calculate the Monte Carlo input phase space parameters at the position of 50 cm (Δz=50 cm) upstream of the isocenter:

(A.4a) $$A_{50}=C_{0}\Delta z^{2}+2B_{0}\Delta z+A_{0}$$

(A.4b) $$B_{50}=C_{0}\Delta z+B_{0}$$

(A.4c) $$C_{50}=C_{0}$$

Bivariate Gaussian surface source defined as input to the Monte Carlo simulations:

(A.5) $$f(x,\theta_{x})=\frac{1}{\sqrt[{\pi }]{A_{50x}C_{50x}-B_{50x}^{2}}}\text{exp}\left\{-\frac{A_{50x}\theta_{xs}^{2}-2B_{50x}x_{s}\theta_{xs}+C_{50x}x_{s}^{2}}{A_{50x}C_{50x}-B_{50x}^{2}}\right\},$$

where the subscript “50x” denotes the phase space parameters in the X direction determined at z=50 cm. The Y diretcion can be represented by a similar expression with the respective set of phase space parameters.

### 2. According to the model implemented in Eclipse

(A.6a) $$\Delta A=\frac{V}{\alpha^{3}E_{0}}\{\alpha^{2}[{\left(S+L\right)}^{2}\phi (\beta_{i})-S^{2}\phi (\beta_{o})]-2\alpha E_{o}[(S+L)\chi (\beta_{i})-S\chi (\beta_{o})]+2E_{0}^{2}(\psi (\beta_{i})-\psi (\beta_{o}))\}$$

(A.6b) $$\Delta B=\frac{V}{\alpha^{2}E_{0}}\{\alpha [S+L]\phi (\beta_{i})-S\phi (\beta_{ο})\}-E_{0}(\chi (\beta_{i})-\chi (\beta_{o}))\}$$

(A.6c) $$\Delta C=\frac{V}{\alpha E_{0}}(\phi (\beta_{i})-\phi (\beta_{o}))$$

where:

ϕ(β)=∫βcβdβ′β′43(1−β′2)43

χ(β)=∫βcβϕ(β′)β′83(1−β′2)32dβ′

ψ(β)=∫βcβχ(β′)β′83(1−β′2)32dβ′

and where α is a material dependent factor used in the “five‐thirds approximation” of the Bethe stopping power equation, *β* is the ratio of proton speed to the speed of light, $E_{0}$ is the proton rest mass, and $\beta_{c}$ is an arbitrary integration constant introduced to avoid singularity at β=0.

Calculation of phase space parameters for any given position and thickness of the RS:

(A.7a) $$A_{RS}(L,S)=A_{OpenBeam}+\Delta A(L,S)$$

(A.7b) $$B_{RS}(L,S)=B_{OpenBeam}+\Delta B(L,S)$$

(A.7c) $$C_{RS}(L,S)=C_{OpenBeam}+\Delta C(L,S)$$

where the subscripts *”RS”* and *”OpenBeam”* refer to the phase space parameters with and without the RS, respectively.
